# Characterization of Blue Mold *Penicillium* Species Isolated from Stored Fruits Using Multiple Highly Conserved Loci

**DOI:** 10.3390/jof3010012

**Published:** 2017-03-01

**Authors:** Guohua Yin, Yuliang Zhang, Kayla K. Pennerman, Guangxi Wu, Sui Sheng T. Hua, Jiujiang Yu, Wayne M. Jurick, Anping Guo, Joan W. Bennett

**Affiliations:** 1 Key Laboratory of Biology and Genetic Resources of Tropical Crops, Ministry of Agriculture, Institute of Tropical Bioscience and Biotechnology, Chinese Academy of Tropical Agricultural Sciences, Haikou 5711001, China; zhangyuliang@itbb.org.cn; 2 Department of Plant Biology and Pathology, Rutgers, The State University of New Jersey, New Brunswick, NJ 08901, USA; k.pennerm@rutgers.edu (K.K.P.); profmycogirl@yahoo.com (J.W.B.); 3 Oak Ridge Institute for Science and Education, Oak Ridge Associated Universities, Oak Ridge, TN 37831, USA; guangxiwu@gmail.com; 4 U.S. Department of Agriculture, Agricultural Research Service , Western Regional Research Center, Albany, CA 94710, USA; sylvia.hua@ars.usda.gov; 5 U.S. Department of Agriculture, Agricultural Research Service, Beltsville, Agricultural Research Center, Beltsville, MD 20705, USA; jiujiang.yu@ars.usda.gov (J.Y.); wayne.jurick@ars.usda.gov (W.M.J.)

**Keywords:** blue molds, stored fruits, *Penicillium* spp., ITS, *benA*, *CaM*

## Abstract

*Penicillium* is a large genus of common molds with over 400 described species; however, identification of individual species is difficult, including for those species that cause postharvest rots. In this study, blue rot fungi from stored apples and pears were isolated from a variety of hosts, locations, and years. Based on morphological and cultural characteristics and partial amplification of the β-tubulin locus, the isolates were provisionally identified as several different species of *Penicillium*. These isolates were investigated further using a suite of molecular DNA markers and compared to sequences of the ex-type for cognate species in GenBank, and were identified as *P. expansum* (3 isolates), *P. solitum* (3 isolates), *P. carneum* (1 isolate), and *P*. *paneum* (1 isolate). Three of the markers we used (ITS, internal transcribed spacer rDNA sequence; *benA*, β-tubulin; *CaM*, calmodulin) were suitable for distinguishing most of our isolates from one another at the species level. In contrast, we were unable to amplify RPB2 sequences from four of the isolates. Comparison of our sequences with cognate sequences in GenBank from isolates with the same species names did not always give coherent data, reinforcing earlier studies that have shown large intraspecific variability in many *Penicillium* species, as well as possible errors in some sequence data deposited in GenBank.

## 1. Introduction

“Blue mold” is a common term used to describe several species of *Penicillium* that cause postharvest decay of important fruit crops because visible sporulation on infected fruits is blue-green in color [1,2]. *Penicillium expansum* [3,4], *Penicillium digitatum* [5], and *Penicillium italicum* [6] not only cause fruit decay and economic losses of apple and citrus fruits in the United States, but also produce extrolites (secondary metabolites) that may be harmful to humans.

*Penicillium* is one of the largest and most important genera of microscopic fungi, with over 400 described species distributed worldwide [7]. Its name comes from the Latin “penicillus”, which refers to the brush-like appearance of the conidiophores that resemble a painter’s brush. The type species for the genus, *P. expansum*, is primarily responsible for postharvest decay of pome fruits [8]. *Penicillium* species are difficult to distinguish from each other (even to expert taxonomists), and many species display a great deal of intraspecific variability [8,9,10,11].

Traditional identification of *Penicillium* species focuses on the color and texture of colonies; the growth rate and size of colonies on standardized media; conidiophore morphology, including branching patterns and shapes, dimensions, and ornamentations of the different parts of the conidiophore; and the production of certain extrolites. However, these morphological and biochemical characteristics may be influenced or changed by various environmental factors, confounding both identification and taxonomic classification [12,13]. Molecular methods are required for unambiguous identification of *Penicillium* species, but the selection of appropriate markers for use in the genus is challenging [14]. The internal transcribed spacer rDNA sequence (ITS) is the most widely sequenced marker for fungi. Universal primers are available, and it is the official sequence for barcoding [15]. Unfortunately, the ITS sequence is not diagnostic enough in *Penicillium* species for distinguishing all closely-related species [15,16]. Furthermore, GenBank contains many misidentified sequences, further complicating *Penicillium* species identification [17]. Because of the limitations associated with the ITS, several additional gene regions including β-tubulin (*benA*), calmodulin (*CaM*), and RNA polymerase II (RPBs) have been used to distinguish closely-related *Penicillium* species [7,10,11,18]. 

Ease, practicality, and accuracy are essential for the identification of *Penicillium* species in agricultural settings in order to predict patterns of virulence, mycotoxin production, and fungicide resistance. Thus, it is important to determine which molecular markers should be used for blue mold verification. The immediate objective of this study was to use molecular markers to verify species identification of blue molds accomplished via traditional approaches, and to compare our sequence data to those published for cognate species in GenBank. The overarching goal of our work is to find a rapid, convenient, and accurate way to identify *Penicillium* species involved in blue mold so as to tailor appropriate strategies for the control of postharvest fruit decay.

## 2. Materials and Methods

### 2.1. Penicillium Isolation, Morphological Identification, and Mycotoxin Detection

Eight *Penicillium* strains were isolated from “Golden Delicious” and “Red Delicious” apples with blue mold in 2011 and 2012 from commercial storage facilities. For comparison, one *P*. *solitum* strain (NJ1) originally obtained as a subculture of a sector of strain 2159A from the Northern Regional Research Laboratory culture collection [19], and one strain of *P. sclerotiorum* (113) collected from a flooded home in Manasquan, New Jersey were included [20,21]. Isolates were purified by culturing from single spores, and a preliminary characterization was conducted using macro- and microscopic observations and measurements, including colony color, diameter and texture, conidiospores and conidiophore morphology, and mycotoxin production. If a culture contained more than one type of spore or colony morphology, isolates were sub-cultured on 2% malt extract agar until the characteristics were consistent. Using identification keys from previous studies [11,22], most of the isolates were tentatively identified to the species level. *Penicillium* isolates that could not be identified microscopically were grouped into morphotypes by their growth characteristics on three differential media: malt extract agar (MEA), Czapek yeast agar (CYA), and corn meal agar (CMA), yeast extract sucrose agar (YES), and creatine sucrose agar (CREA) [7,20,23,24,25]. To obtain a preliminary analysis of mycotoxins (patulin and citrinin), the isolates were cultured in potato dextrose broth at 25 °C for seven days and extracted using an organic solvent extraction method [26,27]. A total of 20 mL potato dextrose broth from growing cultures was extracted three times with 25 mL of ethyl acetate by shaking vigorously for 1 min each time. Then, the organic phases were combined. Five drops of glacial acetic acid were added to the combined organic phase solution, and the solution was evaporated to dryness in a 40 °C water bath under a gentle stream of nitrogen gas. The dried residue was immediately dissolved in 1 mL of acetic acid buffer solution. Acetate buffer was prepared by adding 0.45 mL acetic acid glacial and 0.245 g of sodium acetate trihydrate to 40 mL of ddH_2_O. The pH was adjusted to 4.0 with acetic acid glacial. The volume was then adjusted to 50 mL with ddH_2_O. The toxins in the extracted sample were subjected to thin layer chromatography [26,27].

### 2.2. DNA Extraction and PCR Amplification

For each strain, a culture was grown on potato dextrose agar at 25 °C for 3 days before subculturing to a 250 mL Erlenmeyer flask containing 100 mL liquid potato dextrose medium for 3–4 days with shaking (180–200 rpm) at 25 °C. All the mycelia were separated from the media by filtration through sterile No. 5 Whatman filter paper and transferred to Eppendorf 1.5 mL tubes. These mycelial samples were stored at −70 °C before use. Fungal mycelia were disrupted using a TissueLyser II (Qiagen Inc, West Chester, PA, USA) with beads (5 mm diameter) set at a shaking frequency of 20 Hz per second for 25 min. Then, DNA was extracted using a DNeasy Plant Mini Kit following the manufacturer’s instructions (Qiagen Inc). The purity and concentration of fungal genomic DNA (including mitochondrial DNA) was measured using a NanoDrop spectrophotometer (Thermo Fisher Scientific Inc, Wilmington, DE, USA). The extracted DNA samples had A260/280 and A260/230 values greater than 1.8.

For molecular identification of *Penicillium* species, primers specific for ITS, *benA*, *CaM* and *RPB2* loci were selected for PCR amplification (Table 1) [7]. PCR was performed in a 20 µL reaction system including 1 µL gDNA template (about 100 ng), 0.2 µL Pfu DNA polymerase (2.5 U/µL) (Stratagene), 0.5 µL each forward and reverse primers (10 µM), 1 µL dNTPs (2.5 mM), and added sterile ddH_2_O to a final volume of 20 µL. The thermocycler was programed as follows: pre-heated at 94 °C for 5 min, 35 cycles of denaturation at 94 °C for 45 s, annealing at 55 °C (ITS; *benA*, β-tubulin; and *CaM*, calmodulin) for 45 s, and extension at 72 °C for 1 min, with a final extension at 72 °C for 10 min. For *RPB2* amplification, we used a touch-up PCR according to the method of Visagie and his colleagues [7]. Eight microliters of PCR product was mixed with 1 µL GoldView dye for electrophoresis on a 1.5% agarose gel at 120 V for 15 min. PCR products were cleaned using ExoSAP-IT (Affymetrix, Santa Clara, CA, USA), followed by sequencing using the BigDye sequencing protocol (Applied Biosystems, Inc., Foster City, CA, USA). DNA sequencing was performed at Major Biosystem (Shanghai, China).

### 2.3. Sequencing and Phylogenetic Analysis

Each isolate was identified by DNA sequencing according to a standard protocol [34]. Final sequences were aligned and analyzed using BioEdit 7.2.5 software (http://www.mbio.ncsu.edu/bioedit/bioedit.html) and Clustal Omega [35]. The corresponding fungal isolates were assigned species names after comparison with representative ex-type sequences which were available in NCBI GenBank. All the sequences from this study were deposited in GenBank.

The DNA sequences of the ITS, *benA*, and *CaM* genes from nine of our *Penicillium* isolates and nine other ex-type *Penicillium* isolates (*P*. *griseofulvum* CBS185.27, *P*. *carneum* CBS468.95, *P*. *paneum* CBS303.97, *P*. *sclerotiorum* CV0934, *P*. *expansum* CV2861, *P*. *solitum* FS06278, *P*. *solitum* BT-18-1, *P*. *paneum* CBS464.95, *P*. *sclerotiorum* FS50, and two *Aspergillus* species (*A. flavus* PW2962 and *A. niger* 13L06I1) in GenBank were chosen for phylogenetic analysis. All ambiguous positions were removed. Sequence similarities were inferred using the neighbor-joining method [36]. The percentage of replicate trees in which the associated taxa clustered in the bootstrap test (500 replicates) are shown next to the nodes [37]. The evolutionary distances were computed using the Kimura 2-paramenter method [38] and expressed in units of the number of base substitutions per site. All positions containing gaps and missing data were eliminated. Analyses were conducted using MEGA6 [39].

## 3. Results

### 3.1. The Biological Characteristics of Penicillium spp.

Eight blue mold *Penicillium* spp. isolates from stored fruits were selected based on their morphology in culture, their abilities to produce different toxins, and diversity with respect to host and location from which the isolates were obtained. The isolate number, source, toxin production, and culture characteristics are listed in Table 2. Using traditional means, the eight strains were identified tentatively as belonging to four different *Penicillium* species: *P*. *expansum* (three isolates; R19, R21, and R27), *Penicillium solitum* (three isolates; RS1, SA and NJ1), *Penicillium carneum* (one isolate; G2), and *Penicillium paneum* (one isolate; G9). In our preliminary mycotoxin analysis, *P. expansum* isolates R19 and R27 produced patulin and citrinin, while R21 produced only patulin. For *P*. *solitum* RS1, SA, and NJ1, none of the isolates produced detectable mycotoxin. One isolate of *P*. *sclerotiorum* isolated from an indoor environment source was included for comparison.

### 3.2. Sequencing Analysis of Penicillium spp. and Phylogenetic Analysis

Six sets of primers (Table 1) [7] were chosen to amplify five marker genes from the nine *Penicillium* isolates. Using these primers, not all strains yielded successful PCR products. For example, no sequence from *P*. *expansum* R19 was amplified by the ITS primers; *P. expansum* R21 and *P. solitum* SA were not amplified by the *benA* primers. *P*. *solitum* SA and RS1 were not amplified by *RPB2-1* and *RPB2-2* primers, nor were *P*. *expansum* R19 and R27. All successfully amplified sequences were submitted to GenBank, and their accession numbers are listed in Table 3. The initial species identifications based on traditional criteria were largely confirmed by sequence analysis.

**Figure 1:**
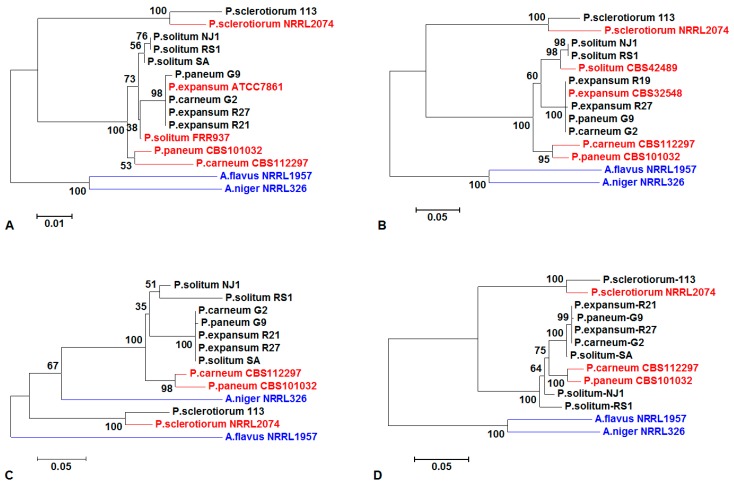
*Penicillium* spp. phylogenetic trees constructed using markers (**A**) ITS; (**B**) *benA*; (**C**) *CaM*; and (**D**) combinations of the two or three genes. Nine of our isolates and nine ex-types from NCBI GenBank were phylogenetically arranged using available marker sequences. The nine strains obtained from NCBI were *P*. *expansum* ATCC7861 = *P*. *expansum* CBS32548, *P*. *carneum* CBS112297, *P*. *paneum* CBS101032, *P*. *sclerotiorum* NRRL2074, *P*. *solitum* CBS42489 = *P*. *solitum* FRR937, *A. flavus* NRRL1957, and *A. niger* NRRL326. All the *Penicillium* species from GenBank are shown in red; the two *Aspergillus* species are shown in blue; our own sequenced *Penicillium* species are shown in black. Three genes were used to perform sequences analysis in Figure 1D, except only two genes (ITS and *CaM*) were used in *P*. *expansum* R21 and *P*. *solitum* SA analysis.

Phylogenetic trees were made with each individual gene: ITS (Figure 1A), *benA* (Figure 1B), and *CaM* (Figure 1C), and where successful amplification product has been achieved by all three combined (Figure 1D) [40,41]. Because so few strains were amplified by the *RPB2-1* and *RPB2-2* primers, these sequences were not used in the phylogenetic analysis. In building the phylogenetic tree, the equivalent sequences for ITS of the ex-type of *P*. *solitum* and *P*. *expansum* in GenBank have the culture collection numbers FRR937 and ATCC7861, respectively; however, the *benA* sequences of *P*. *solitum* and *P*. *expansum* are found to have the culture collection numbers CBS42489 and CBS32548, respectively, which are transfers of the same strains. There were no available *CaM* sequences of the ex-types of *P. expansum* and *P. solitum* in GenBank. All the *P. expansum* and *P. solitum* species were grouped in one clade; however, not all isolates with the same tentative species identification could be grouped together in a phylogenetic tree. The strains tentatively identified as *P*. *carneum* G2 and *P*. *paneum* G9 grouped with *P. expansum* when analyzed by ITS, *benA*, and *CaM* (Figure 1). *Penicillium sclerotiorum* isolates were consistently grouped together using all three markers. *Aspergillus flavus* and *A. niger* were used as outgroups, and were also grouped together using all markers except for *CaM* (Figure 1). When a multi-gene (ITS, *benA*, and *CaM*) phylogeny was constructed based on all three genes, the topologies for strains supposedly in the same species did align and form the same clades, except *P*. *carneum* G2 and *P*. *paneum* G9 (Figure 1D). 

### 3.3. Mycotoxin

We also did a preliminary screen for the production of patulin and/or citrinin in some *Penicillium* isolates. Two of the three identified *P. expansum* strains produced both toxins. *P*. *expansum* R21 produced patulin, but did not produce a detectable amount of citrinin. None of the *P*. *solitum* strains (RS1, SA, and NJ1) appeared to produce either of the toxins. If *P*. *carneum* G2, *P*. *paneum* G9, or *P*. *sclerotiorum* 113 produce mycotoxins, they may produce toxin in trace amounts (Table 2). 

## 4. Discussion and Conclusions

In storage, harvested crops and fruits are subjected to various attacks from insects and microbial plant pathogens. *Penicillium* species are a major cause of storage decays in pome and citrus fruits, as well as other postharvest crops [6,11,42,43,44]. It is often difficult to identify and distinguish different *Penicillium* species, as the genus is large and many of the common species look similar to each other. However, accurate identification is necessary for effective decay and toxin control, to estimate fungicide resistance, and to tailor control strategies. For example, *P. expansum* is the most virulent species and produces more toxins on apples and pears than *P*. *solitum* [45], making it important to have a fast and reliable method for distinguishing between the two species. Considering the frequently ambiguous identification of blue mold fungi, molecular tools are needed for the correct identification of closely-related *Penicillium* species [7,18]. In this study, we identified eight *Penicillium* isolates collected from fruits with blue mold by traditional means. We then used molecular markers to compare our sequences to sequences of the ex-type strains to confirm the species identifications and further to characterize the isolates isolated from stored fruits.

The ITS regions of ribosomal DNA have been widely applied in phylogenetic studies of fungal genomes because these regions are highly conserved and can be easily investigated using PCR amplification [46]. However, the ITS sequence information alone cannot be used to place a fungus at the species level. In this study, using the ITS1F-ITS4 primer set, we could easily amplify divergent ITS regions among morphologically distinct fungal species, but we could not rely on these sequences alone to differentiate closely-related *Penicillium* species. Other markers are needed in combination with the ITS markers for effective fungal identification. 

We attempted to employ other common molecular markers to more precisely distinguish *Penicillium* isolates [47]. These included the genes of *β-tubulin*, *calmodulin*, and *RPB2*, all of which have been previously shown to be effective markers for species identification in other fungal genera such as *Alternaria* [48], *Aspergillus* [49,50], *Botrytis* and *Fusarium* [51], and *Ramularia* and *Wallemia* [52]. The β-tubulin marker locus includes about 550 bp of the 5′ end of the gene with introns and exons 3, 4, 5, and partial exon 6. The calmodulin marker is about 580 bp long, containing introns and exons 2, 3, 4, and partial exon 5. The *RPB2* markers involve about 1,000 bp of the gene for RNA polymerase II second largest subunit [33]. At least with respect to the primers we used, these markers were not applicable to all of the tested *Penicillium* isolates from stored fruits. The primers for *RPB2* only facilitated PCR amplification for half of the isolates.

One goal of studying conserved sequences within the genus *Penicillium* is the possibility of developing a DNA array representing all possible blue mold pathogens. Such an array would be useful in the rapid diagnoses of strains that have developed resistance to benzimidazole fungicides [53]. Another goal is to improve our understanding of how mycotoxin production is related to the pathogenicity and virulence of different strains and species of *Penicillium*. Some *Penicillium* mycotoxins—especially patulin—are toxic to humans and animals, and contaminate food products [54,55]. In this study, we are not sure if *P. carneum* G2, *P. paneum* G9, or *P. sclerotiorum* 113 could produce mycotoxins or not, and recommend that different agar media should be used to detect their production. In future work with *Penicillium* species identification and genome sequencing, we are developing other more effective markers, with an emphasis on distinguishing the most virulent and toxigenic blue molds that cause postharvest decay.

Finally, high genome variation and the existence of two different mating type genes were recently shown in *P*. *expansum*—a species previously considered to lack a sexual stage [56]. Future studies on *Penicillium* strains involved in blue mold decays need to consider the fact that phenotypic variability in virulence, mycotoxin production, and fungicide resistance in the *P*. *expansum* species complex may be due to occult sexual recombination [56].

## Figures and Tables

**Table 1:** Primers used for *Penicillium* species identification.

Gene	Primer	Sequence (5′→3′)	Length (bp)	Reference
Internal transcribed spacer (ITS)	ITS1F	CTTGGTCATTTAGAGGAAGTAA	~600	[28]
	ITS4	TCCTCCGCTTATTGATATGC		
β-tubulin (*benA*)	Bt_2_a	GGTAACCAAATCGGTGCTGCTTTC	~550	[29]
	Bt_2_b	ACCCTCAGTGTAGTGACCCTTGGC		
Calmodulin (*CaM*)	CMD5	CCGAGTACAAGGARGCCTTC	~580	[30]
	CMD6	CCGATRGAGGTCATRACGTGG		
	CF1	GCCGACTCTTTGACYGARGAR	~750	[31]
	CF4	TTTYTGCATCATRAGYTGGAC		
RNA polymerase II second largest subunit (*RPB2-1*)	5F	GAYGAYMGWGATCAYTTYGG	~1000	[32]
	7CR	CCCATRGCTTGYTTRCCCAT		
RNA polymerase II second largest subunit (*RPB2-2*)	5Feur	GAYGAYCGKGAYCAYTTCGG	~1000	[33]
	7CReur	CCCATRGCYTGYTTRCCCAT		

**Table 2:** Cultural characterization and mycotoxin production of nine blue mold *Penicillium* isolates.

Species	Host	Source (Location)	Year	Patulin ^1^	Citrinin	Back Color
*P. carneum* G2	Golden delicious	Pennsylvania	2011	+/−	+/−	tan
*P. paneum* G9	Golden delicious	Pennsylvania	2011	+/−	+/−	yellow
*P. expansum* R19	Red delicious	Pennsylvania	2011	+	+	tan
*P. expansum* R21	Red delicious	Pennsylvania	2011	+	−	green
*P. expansum* R27	Red delicious	Pennsylvania	2011	+	+	green
*P. solitum* RS1	Apple	Oregon	2011	−	−	tan
*P. solitum* SA	Peach seed	West Virginia	2011	−	−	tan
*P. solitum* NJ1	-	NRRL, Illinois	2012	−	−	tan
*P. sclerotiorum* 113	Obtained from home living room	New Jersey	2013	+/−	+/−	orange

^1^ The mycotoxins patulin and citrinin were detected by organic solvent extraction followed by thin layer chromatography (TLC) separation and visualization under UV light at 365 nm wave length; +/−: low level faint band on TLC plate and it is unclear whether the mycotoxin is produced or not; +: mycotoxin present; −: mycotoxin not detected; NRRL = culture collection at National Center for Agricultural Utilization Research located in Peoria, Illinois, USA.

**Table 3:** Accession numbers of amplified nucleotide sequences from *Penicillium* spp. isolates.

*Penicillium* spp.	ITS	benA	CaM ^a^	CaM ^b^	RPB2-1	RPB2-2
*P. carneum* G2	KX243324	KX243333	KX243341	-	KX243353	KX243356
*P. paneum* G9	KX243325	KX243334	KX243342	-	KX243354	KX243357
*P. expansum* R19	-	KX243337	-	-	-	-
*P. expansum* R21	KX243328	-	KX243345	KX243351	KX243355	KX243358
*P. expansum* R27	KX243329	KX243338	KX243346	-	-	-
*P. solitum* SA	KX243330	-	KX243347	-	-	-
*P. solitum* RS1	KX243331	KX243339	KX243348	-	-	-
*P. solitum* NJ1	KX243323	KX243332	KX243340	-	KX243352	-
*P. sclerotiorum* 113	KX365203	KX365204	KX365205	-	KX365206	-

“-” denotes no clear PCR products were obtained using primers from Table 1. *P. sclerotiorum* 113 was added for comparison; ^a^ *CaM*: amplified using CMD5 and CMD6 primers; ^b^ *CaM*: amplified using CF1 and CF4 primers, *RPB2-1* amplified using 5F and 7CR primers; *RPB2-2* using 5Feur and 7CReur primers. See Table 1.
